# Licochalcone A Inhibits *BDNF* and *TrkB* Gene Expression and Hypoxic Growth of Human Tumor Cell Lines

**DOI:** 10.3390/ijms21020506

**Published:** 2020-01-13

**Authors:** Michitsune Arita, Junichi Koike, Nobuji Yoshikawa, Motonari Kondo, Hiromichi Hemmi

**Affiliations:** 1 Department of Molecular Immunology, Toho University School of Medicine, Tokyo 143-8540, Japan; hhemmi@med.toho-u.ac.jp; 2 Department of Surgery, Toho University School of Medicine, Tokyo 143-8540, Japan; jkoike18@med.toho-u.ac.jp; 3 Cokey Systems, Co., Ltd., Tokyo 102-0075, Japan; yoshikawa@cokey.co.jp

**Keywords:** BDNF/TrkB, hypoxia, licochalcone A, human established cell lines, transcriptional inhibitor

## Abstract

Hypoxic cellular proliferation is a common feature of tumor cells and is associated with tumor progression. Therefore, the inhibition of hypoxic cellular proliferation is expected to regulate malignancy processes. Licochalcone A (LicA) is known to show inhibitory effects on cell growth in normoxia, but it is unclear whether LicA exerts similar effects in hypoxia. Here, we studied the inhibitory activity of LicA in the hypoxic cellular proliferation of tumor cells and its molecular mechanism using human cell lines derived from various tumors including neuroblastoma and colorectal cancer. LicA inhibited cell growth at a 50% inhibitory concentration between 7.0 and 31.1 µM in hypoxia. LicA significantly suppressed hypoxic induction of tropomyosin receptor kinase B (*TrkB*) gene expression at the transcription level. LicA also downregulated mRNA levels of the TrkB high-affinity ligand brain-derived neurotrophic factor, but not neurotrophin-4, another TrkB ligand, or glyceraldehyde-3-phosphate dehydrogenase, indicating that the inhibitory activity of LicA is selective. Since both LicA-treatment and *TrkB*-knockdown decreased activation of protein kinase B in hypoxia, LicA exerts its inhibitory effect against hypoxic cell growth through inhibition of the TrkB-AKT axis. These results suggest that LicA has therapeutic potential for malignant tumors including neuroblastoma and colorectal cancer.

## 1. Introduction

Hypoxic adaptation, which allows cellular proliferation and survival even at low oxygen levels, is a common feature of tumor cells and promotes tumor malignancy. To adapt to a hypoxic microenvironment, cells change the expression of the genes responsible for extracellular neo-vascularization and the intracellular metabolic shift to aerobic glycolysis [1,2]. The metabolic shift in hypoxia causes acidosis and induces cell invasion and metastasis via epithelial-to-mesenchymal transition (EMT) [3,4]. A key regulator of these processes is the phosphatidylinositol 3-kinase/protein kinase B/mammalian target of rapamycin (PI3K/AKT/mTOR) signaling pathway [1,5,6].

The PI3K/AKT/mTOR pathway is initiated by binding of ligands to receptor tyrosine kinases (RTKs) [1,6]. Tropomyosin receptor kinase B (TrkB), an essential signaling initiator for neurodevelopment, is one of the tumor-associated RTKs [7,8]. TrkB and its high-affinity ligand brain-derived neurotrophic factor (BDNF) had originally been recognized as notable markers for poor prognosis in neuroblastoma and as key players for its progression [9,10,11]. Recently, TrkB and its ligand, BDNF, have been shown to be overexpressed in various types of cancer, such as cervical, breast, and colorectal cancer (CRC), and to be associated with their poor prognosis [7,12,13]. TrkB has also been shown to be a key player in metastasis in studies using a rat intestinal epithelial cell line [14], a mouse xenograft model [15], and human lung cancer cell lines [16]. Furthermore, TrkB is upregulated in hypoxia [16,17]. Thus, hypoxic induction of BDNF/TrkB is expected to be an event that may serve as a therapeutic target for preventing the progression of advanced cancer, especially neuroblastoma and CRC.

Licochalcone A (LicA), a *Glycyrrhiza* flavonoid, has been shown to have anti-tumor activity in various systems. Potential therapeutic effects of LicA have been demonstrated in gastric, lung, and cervical cancer cells [18,19,20,21]. Although LicA has been reported to inhibit the activation of TrkB-downstream molecules, such as PI3K, AKT, and mTOR in previous studies, the effects of LicA on TrkB and BDNF as well as on hypoxic cellular proliferation are not yet known.

In this study, we examined the inhibitory effect of LicA on hypoxic growth in human cell lines derived from various tumors, including neuroblastoma and CRC. Furthermore, we examined the effect of LicA on TrkB/BDNF, an initial point of hypoxic cellular survival and proliferative signaling pathways, to explore the mechanism underlying LicA-induced growth inhibition.

## 2. Results

### 2.1. LicA Inhibits Cell Growth in Hypoxia

**Figure 1:**
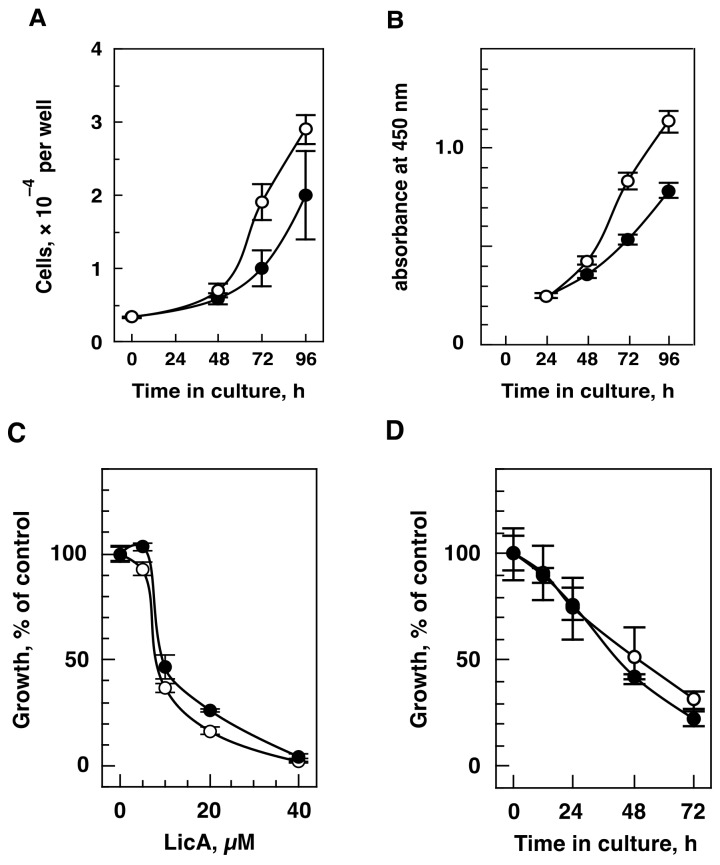
Growth inhibitory activity of Licochalcone A (LicA). (**A**) Normoxic (open circles) and hypoxic (closed circles) proliferative ability of SK-N-SH cells assessed by cell counts. Cells (5 × 10^3^ cells/well of a 12-well plate) were pre-cultured in normoxia for 24 h and cultured in normoxia and hypoxia. The number of cells was counted at the indicated time. (**B**) Normoxic (open circles) and hypoxic (closed circles) proliferative ability of SK-N-SH cells assessed by mitochondrial activity. Cells (6 × 10^2^ cells/well of a 96-well plate) were cultured in normoxia and hypoxia. Cell growth was monitored using WST-8 assays at the indicated time. (**C**) Dose dependency. SK-N-SH cells (3 × 10^3^ cells/well) were cultured in hypoxia (closed circles) and normoxia (open circles) with the indicated concentrations of LicA for 72 h. Cell growth was determined as described in (**B**). (**D**) Time course of inhibition. SK-N-SH cells (3 × 10^3^ cells/well) were cultured in hypoxia (closed circles) and normoxia (open circles) with 20 µM LicA for the times indicated. Cell growth was determined as described in (**B**).

We studied the effects of LicA on hypoxic and normoxic cell growth using the neuroblastoma SK-N-SH cell line, which showed somewhat lower proliferative ability in hypoxia than in normoxia based on cell counts (Figure 1A) and mitochondrial activity (Figure 1B). LicA at concentrations of 5–40 µM showed growth inhibitory activity in hypoxia-exposed cells at 72 h (Figure 1C). LicA (20 µM) exerted an inhibitory activity in a time-dependent manner (Figure 1D). Significant growth inhibition against pre-treatment (0 h) was detected at 24 h (*P* value for normoxia and hypoxia are 0.0385 and 0.0048, respectively) and continued for 72 h. The 50% inhibitory concentration (IC_50_) for LicA was 8.4 µM (Table 1). LicA inhibition seems to be similar in hypoxic and normoxic cells.

Since the growth inhibitory activity of LicA was observed in neuroblastoma SK-N-SH cells, we examined the effect on six other cell lines derived from various origins including neuroblastoma, CRC, and cervix carcinoma, as listed in Table 1. All of the cell lines showed a proliferative ability in hypoxia as observed for SK-N-SH (Table 1). In addition to the effects seen in SK-N-SH cells, LicA showed potent inhibitory activity in the colorectal SW480 and SW620 cell lines and the cervix HeLa S3 cell line, with IC_50_ values between 7.0 and 10.7 µM (Table 1). The IC_50_ values of the other cell lines examined were approximately 3-fold (24.9–31.1 µM). LicA exhibited inhibitory activity against hypoxic cellular proliferation regardless of tumor origin, even though IC_50_ values varied. Since SK-N-SH is one of the most sensitive cell lines to LicA, we used this cell line in the subsequent investigations.

### 2.2. LicA Inhibits Hypoxic Activation of TrkB and BDNF Expression

**Figure 2:**
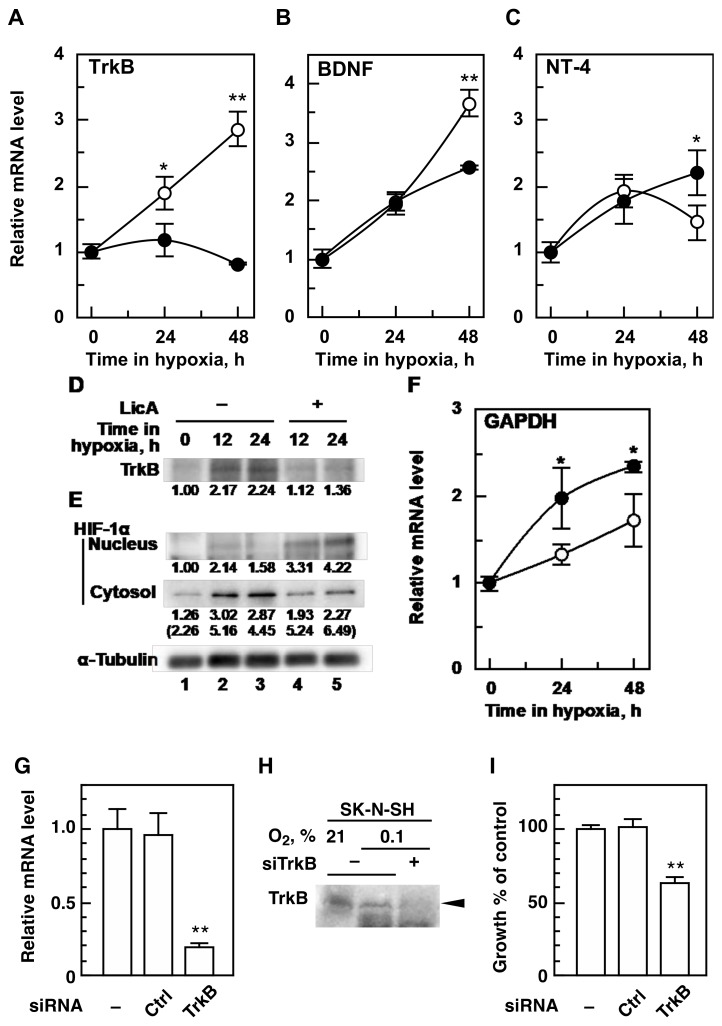
Inhibition of TrkB and brain-derived neurotrophic factor (BDNF) expression in SK-N-SH. (**A**–**C**,**F**) Cells (2 × 10^4^ cells/well) were cultured in hypoxia for the indicated periods with 20 µM LicA (closed circles) and without (open circles), and total RNA was isolated. mRNA levels were determined by quantitative reverse-transcription polymerase chain reaction (qRT-PCR). (**D**) Inhibition of TrkB at the protein level by 20 µM LicA was determined by immunoblot. (**E**) A specific band of HIF-1α in the cytosol and nuclear fractions of cells treated with or without 20 µM LicA in hypoxia was detected by immunoblot. (**G**) Cells (2 × 10^5^ cells/well of a 24-well plate) were cultured for 24 h in normoxia and transfected with siRNA. Cells were further cultured for 24 h in hypoxia and total RNA was isolated. mRNA levels were determined as described above. (**H**) Cells (1 × 10^6^ cells/10 cm dish) were cultured and transfected with siRNA as described in (**G**). Inhibition of TrkB at the protein level by TrkB knockdown was determined by immunoblot. (**I**) Cells (3 × 10^3^ cells/well of a 96-well plate) were cultured and transfected with siRNA as described in (**G**). After 48 h culture in hypoxia, cell growth was determined as described in Figure 1B. * *p* < 0.05, ** *p* < 0.01.

Proliferative signals in neuroblastoma cells are known to be initiated by ligand-binding to TrkB [22,23] (reviewed in [24,25]). To determine whether TrkB and its high-affinity ligand BDNF increase in hypoxia and whether LicA causes a decrease in either TrkB or BDNF, we focused on investigating the effects of LicA on the expression of the *TrkB* and *BDNF* genes. As expected, *TrkB* mRNA levels in SK-N-SH cells were increased by hypoxia in a time-dependent manner, and they were increased 2.9-fold of those seen in normoxic cells at 48 h (Figure 2A). Hypoxic induction of *TrkB* mRNA levels was significantly inhibited to a basal level by 20 µM LicA detected at 24 h or 48 h (Figure 2A). A similar inhibitory activity of LicA was confirmed at the TrkB protein level, which was increased approximately 2-fold by hypoxia (Figure 2D). Inhibition by LicA was detected at 12 h, and LicA-treated cells showed 51.6% and 60.7% of the band intensity of untreated cells at 12 h and 24 h, respectively. *BDNF* mRNA levels were also increased 3.7-fold at 48 h by hypoxia in a time-dependent manner (Figure 2B). *BDNF* mRNA levels were also significantly inhibited by LicA, but this effect was detected only after 48 h and appeared to be weaker than that on *TrkB*. We also monitored the mRNA level of neurotrophin-4 (*NT-4*), a low affinity ligand for TrkB. While hypoxia transiently increased *NT-4* mRNA levels at 24 h (1.9-fold) and decreased *NT-4* mRNA levels at 48 h (1.4-fold), LicA continuously increased hypoxic *NT-4* mRNA levels significantly at 48 h (2.1-fold; Figure 2C) without any inhibition. Taken together, LicA does not suppress universal mechanisms such as bindings of general transcription factors or RNA polymerization but specific mechanisms for *TrkB* and *BDNF* gene expression.

TrkB as well as BDNF have been reported to be overexpressed in various types of cancer, such as colorectal and cervical cancers, and BDNF/TrkB overexpression has been shown to confer a migratory phenotype and chemotherapy resistance to tumor cells [12]. To determine whether the inhibitory activity of LicA on TrkB and BDNF expression in hypoxia is specific to the SK-N-SH cell line or if it is also exerted in cell lines from other neuroblastoma and/or tumors, we tested the effects of LicA on the amounts of *TrkB* and *BDNF* mRNA in the cell lines listed in Table 1. Various levels of *TrkB* mRNA were detected in the cell lines we tested compared to *TrkB* mRNA levels in SK-N-SH when cells were cultured in hypoxia for 48 h (Table 2). LicA at a concentration of 20 µM inhibited hypoxic *TrkB* mRNA expression in all cell lines tested with a wide range of inhibitory activity (47.4% to 94.8%; Table 2). The effects of LicA on *BDNF* expression were examined in three other cell lines: neuroblastoma TGW, colorectal SW480, and cervical HeLa S3. Hypoxic *BDNF* expression in these cell lines was approximately 3.8-to 10-fold that of SK-N-SH. LicA inhibited *BDNF* expression by 13.7% to 96.6% (Table 2). Thus, the inhibitory activity of LicA may be seen generally in tumor cells which overexpress TrkB and BDNF.

*TrkB* and *BDNF* were upregulated in hypoxia, and LicA inhibited the upregulation, as shown in Figure 2A. This result suggests that LicA suppresses transactivators or induces repressors. To ascertain whether LicA suppresses hypoxia-inducible transcription factor-1 (HIF-1), a key transactivator in hypoxia, we monitored the protein levels of the HIF-1 α-subunit (HIF-1α), an oxygen-sensitive subunit of HIF-1, in the cytosol and nucleus. We found that nuclear levels of HIF-1α were enhanced by LicA, whereas cytosolic levels were decreased (Figure 2E), indicating that LicA likely activates HIF-1 by increasing the nuclear transport of HIF-1α. Consistently, hypoxic expression of the glyceraldehyde-3-phosphate dehydrogenase (*GAPDH*) gene, known to also be upregulated by HIF-1, was increased by LicA (Figure 2F). These results indicate that LicA exhibits inhibitory activity on the hypoxic mRNA levels of *TrkB* and *BDNF* through a mechanism other than HIF-1 suppression. Further studies are needed to determine the target molecules of LicA.

The inhibitory effect of LicA on mRNA levels was detected earlier in the *TrkB* gene than in the *BDNF* gene. To determine whether the hypoxic growth inhibition of SK-N-SH by LicA is started by downregulation of the *TrkB* mRNA level, we performed knockdown experiments. *TrkB* was downregulated by siRNA for the *TrkB* gene at the mRNA and protein levels (Figure 2G,H). Under these conditions, hypoxic cell growth of SK-N-SH was significantly inhibited (Figure 2I). These results suggest that LicA exerts its inhibitory effect against hypoxic cell growth of SK-N-SH through the suppression of *TrkB* gene expression at the mRNA level.

### 2.3. LicA Inhibits TrkB-Downstream Cell Growth Signaling

**Figure 3:**
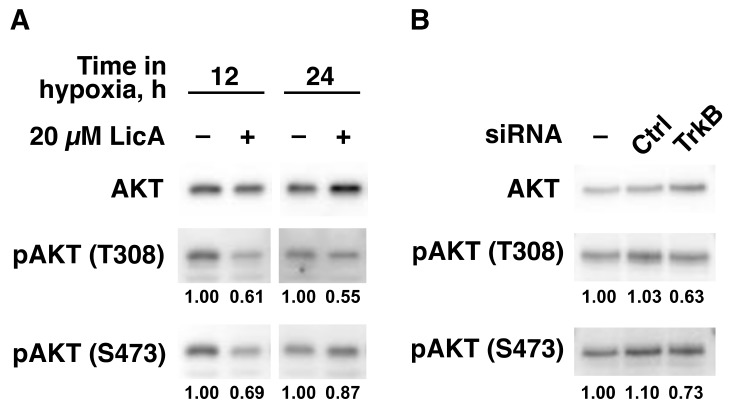
Inhibitions of TrkB-downstream AKT activation in SK-N-SH by LicA. (**A**) Cells (1 × 10^6^ cells/10 cm dish) were cultured in hypoxia for the indicated periods with 20 µM LicA. AKT protein and its phosphorylated forms (pAKT) at Thr308 (T308) and Ser473 (S473) were determined by immunoblot. The ratios of pAKT(T308) and pAKT(S473) levels to AKT level were expressed as relative values to untreated control. (**B**) Cells (2 × 10^5^ cells/10 cm dish) were cultured in normoxia for 24 h and transfected by indicated siRNA in hypoxia for 48 h. The levels of pAKT(T308) and pAKT(S473) proteins were determined by immunoblot and expressed as described in (**A**).

We showed that LicA inhibited hypoxic cell growth of SK-N-SH by downregulation of the mRNA level of *TrkB*, which was known to transduce via downstream pathways including the PI3K/AKT cascade [22,23] (reviewed in [24,25]). To determine whether LicA exhibits its inhibitory effect on SK-N-SH cell growth via inhibition of the TrkB/PI3K/AKT axis, we investigated the effect of LicA on AKT activation regulated by phosphorylation at Thr308 (T308) and Ser473 (S473). LicA inhibited phosphorylation of AKT at both T308 and S473 (Figure 3A). The inhibition was detected at 12 h, which was consistent with the effects of LicA on cell growth (Figure 1D) and on TrkB protein level (Figure 2D). To more directly evaluate whether the inhibition of AKT activation is due to downregulation of TrkB expression, we performed knockdown experiments. The knockdown of *TrkB* using siRNA also downregulated AKT activation at both phosphorylation sites by 38.8% at T308 and 33.6% at S473 against control siRNA (Figure 3B). These results suggest that LicA exhibits growth inhibition through interception of the TrkB/PI3K/AKT cell growth signaling cascade.

### 2.4. LicA inhibits TrkB and BDNF Promoter Activity

**Figure 4:**
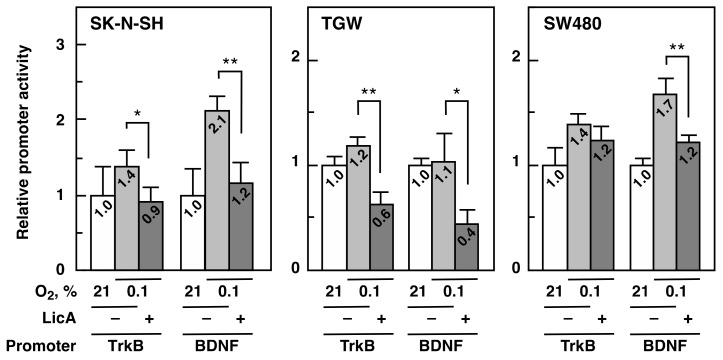
Reporter gene assay for *TrkB* and *BDNF*. The indicated cell lines were transfected with reporter plasmids covering the regulatory regions of the *TrkB* and *BDNF* genes. After 24 h, cells were cultured further in normoxia (open bars) and in hypoxia (0.1% O_2_) without (light-shaded bars) or with 20 µM LicA (dark-shaded bars). After 6 h, the cells were harvested, and luciferase activity was measured. * *p* < 0.05, ** *p* < 0.01.

We observed that LicA selectively downregulated the mRNA levels of *TrkB* and *BDNF* genes. mRNA levels depend on a balance between transcription and mRNA degradation. To determine if LicA inhibits transcription of *TrkB* and *BDNF* mRNA, we constructed reporter plasmids covering the promoter region of each gene and performed reporter gene assays in two neuroblastoma cell lines, SK-N-SH and TGW, and a CRC cell line, SW480. The promoter activities of *TrkB* and *BDNF* were higher in hypoxia than in normoxia. LicA (20 µM) reduced the promoter activity of *TrkB* and *BDNF* in all cell lines (Figure 4). The inhibition of *TrkB* was approximately 34.8% in SK-N-SH, 47.1% in TGW, and 10.9% in SW480. The inhibition of *BDNF* was approximately 46% in SK-N-SH, 57.3% in TGW, and 27.4% in SW480. These results suggest that LicA inhibits *TrkB* and *BDNF* gene expression at the transcriptional level.

## 3. Discussion

LicA blocks cell proliferation in hypoxia as well as in normoxia. LicA also selectively suppresses the hypoxic expression of *TrkB* and *BDNF* at the transcription level. The selectivity of LicA for *TrkB* and *BDNF* might be based on these gene-specific regulatory factors involved in the transcription of the genes such as activators or repressors. HIF-1 is a key transactivator that accumulates in hypoxia and has been reported to upregulate *TrkB* transcription in human neuroblastoma and lung cancer cell lines in hypoxia [16,17]. However, the inhibitory activity of LicA might not be due to suppression of HIF-1, since LicA enhanced nuclear levels of HIF-1α in hypoxia (Figure 2E). We suggest that, for the *TrkB* and *BDNF* genes, LicA may inhibit other transactivating factors, or that it may enhance suppressive factors. LicA also inhibits normoxic *TrkB* expression (our unpublished results), indicating that the factors might play in both normoxia and hypoxia. Based on computational prediction using the JASPAR database [26], several binding sites for transcription factors, such as LMX1B, NOTO, MZF1, MSX2, NKX3-2, and AP-1, are commonly found in the regulatory regions used in the reporter gene assay. Further investigation is required to determine the details of the transcriptional inhibition observed with LicA.

TrkB has been reported to regulate cellular proliferation in various cancer cells (reviewed in [12,27]). In accordance with this notion, *TrkB* knockdown in the SK-N-SH cell line in hypoxia caused inhibition of cellular proliferation in this study (Figure 2I). However, the extent of the growth inhibition achieved by *TrkB* knockdown was less extensive than that seen with LicA. This suggests that LicA is most likely to suppress not only *BDNF* and *TrkB* but also other genes responsible for hypoxic proliferation.

Potential therapeutic effects of LicA have been demonstrated using various human cancer cell lines [18,19,20,21]. In these reports, LicA was shown to exert the effects by inhibiting the activating phosphorylation of signal transducers, such as PI3K, AKT, mTOR, and mitogen-activated protein kinases (MAPKs). In the present study, we show that LicA suppresses expression levels of the *BDNF* and *TrkB* genes and also decreases AKT activation (Figure 3).

In this study, IC_50_ values of LicA varied widely, ranging from 7.0 to 35.5 µM (Table 1). Less sensitive cell lines were shown to have abnormal activation downstream of growth signaling pathways. TGW and GOTO are known to have several hundred *MYCN* gene copies and produce an enormous amount of the MYCN protein [28] downstream of TrkB and mTOR [29]. Activated downstream signaling events may not be enough to detect the inhibitory capability of LicA, indicating that a molecular-targeted compound in combination with LicA might be useful for the treatment of solid tumors.

It has been reported that TrkB activation initiates three downstream signaling cascades, namely PI3K/AKT/mTOR, Ras/extracellular signal-regulated kinase (ERK), and phospholipase C-γ/protein kinase C (PLCγ/PKC) [12,27]. The activating mutations of the K-ras gene constitutively turn on the downstream K-ras-transduced signaling pathways, Ras/ERK and PI3K/AKT. However, two CRC cell lines harboring the G12V K-ras mutation, SW480 and SW620 [30], have been shown to be relatively sensitive to LicA (Table 1). This finding may indicate that the K-ras-transduced pathways are less essential for SW480 and SW620 than the PLCγ/PKC pathway, which is not transduced by K-ras, and that BDNF/TrkB seems to be important for the proliferation of these CRC cell lines. Taken together with previous studies reporting that TrkB overexpression has been observed in advanced CRC [25,31], our results indicate that curbing of hypoxic TrkB and BDNF expression may be a potent therapeutic strategy for certain types of ras-mutated CRCs.

In summary, we demonstrated here that LicA is a novel BDNF/TrkB inhibitor that suppresses gene transcription selectively; therefore, LicA may potentially be a key therapeutic compound for the treatment of malignant tumors including neuroblastoma and CRC.

## 4. Materials and Methods 

### 4.1. Materials

LicA was purchased from Sigma-Aldrich (St. Louis, MO, USA). Rabbit monoclonal antibodies against TrkB (80E3) and phosphorylated AKT at T308 (244F9) and at S473 (193H12) were obtained from Cell Signaling Technology (Danvers, MA, USA). Mouse monoclonal antibodies against HIF-1α (54/HIF-1α) and AKT (55) were obtained from BD Biosciences (San Jose, CA, USA). Mouse monoclonal antibody against α-tubulin (B-5-1-2) was obtained from Sigma-Aldrich. Unless otherwise indicated, reagents of molecular biological or biochemical grades were used.

### 4.2. Cells and Cell Culture

The human neuroblastoma cell line SK-N-SH [32], the human CRC cell lines SW480 and SW620, and the human cervix carcinoma cell line HeLa S3 were obtained from American Type Cell Culture (Rockville, MD, USA). The human neuroblastoma cell lines TGW and GOTO were described previously [33]. All cell lines were maintained in MEM supplemented with 10% fetal bovine serum. The cells were cultured at 37 °C in a humidified atmosphere with 5% CO_2_. Cell passaging was performed weekly. In hypoxia, cells were cultured in 5% CO_2_ with 0.1% O_2_ atmospheric conditions, and the O_2_ concentration was controlled using a ProOx model 110 controller (BioSpherix, Parish, NY, USA) as previously described [34]. The cells were continuously subjected to hypoxia until they were harvested for the subsequent experiments.

### 4.3. Cell Growth Monitoring

Cell growth was monitored using a 2-(2-methoxy-4-nitrophenyl)-3-4-nitrophenyl)-5-(2,4-disulfophenyl)-2H-tetrazolium, monosodium salt (WST-8) kit (Kishida Chemical, Osaka, Japan). Cells were plated in 96-well plates at a density of 3–7 × 10^3^ cells/well in a total volume of 100 µL. After culturing the cells for 24 h in normoxia, various dosages of compounds were added in triplicate, and culturing was continued under either normoxic or hypoxic conditions. After cultivation for the appropriate periods, 10 µL of WST-8 solution was added and the cells were incubated for 4 h at 37 °C. The absorbance at 450 nm was measured using a microplate reader (Model 680; Bio-Rad Laboratories, Hercules, CA, USA) and analyzed.

### 4.4. Quantitative Reverse-Transcription Polymerase Chain Reaction

Gene expression at the mRNA level was quantified by quantitative reverse-transcription polymerase chain reaction (qRT-PCR). Exponentially growing cells were seeded in a 24-well plate at an initial density of 2–4.5 × 10^4^/well and cultured at 37 °C. After 24 h, the plates were placed in a normoxic or hypoxic (0.1% O_2_) chamber. Total RNA was extracted using Isogen-II (Nippon Gene, Tokyo, Japan). A One Step SYBR PrimeScript PLUS RT-PCR kit (Takara Bio, Shiga, Japan) was used for the subsequent cDNA synthesis and RT-PCR. Each sample was amplified in triplicate and analyzed using an Applied Biosystems 7500 Fast Realtime PCR system (Life Technologies, Carlsbad, CA, USA). Ribosomal 18S RNA was used for normalization. The primer sequences used in this study are listed in Table 3.

### 4.5. Immunoblotting

Cells at an initial density of 5 × 10^5^ cells per 10 cm dish were cultured for 24 h under normoxic condition and transferred to a hypoxic chamber (0.1% O_2_). After the indicated hours of hypoxia exposure, the cells were harvested, lysed, and immunoblotting analysis was performed as described previously [37]. For HIF-1α, nucleus and cytosol fractions were prepared as described previously [38]. Proteins in heat-denatured cell lysates were separated by SDS-PAGE and then transferred to PVDF membranes in Tris-Gly buffer. For TrkB, a Tris-lactic acid buffer system [39] was used. To detect proteins, the primary antibodies listed above were used. α-Tubulin was used as a loading control. The blots were visualized using the Amersham ECL-plus system (GE Healthcare UK, Buckinghamshire, UK) and imaged and quantified using a chemiluminescent image analyzer (EZ-Capture MG; Atto, Tokyo, Japan).

### 4.6. siRNAs and Transfection

Cells at an initial density of 2 × 10^5^ cells per 10 cm dish were cultured for 24 h under normoxic conditions and transfected with siRNA by changing the culture medium to Dharmacon Accell siRNA delivery medium (Horizon Discovery, Cambridge, UK) containing 1 µM Accell SMARTpool siRNA (Horizon Discovery, E-003160–01 for TrkB, D-001910-10 for non-targeting control). After 24 h culture in normoxia, the Accell siRNA delivery medium was changed to culture medium and cells were further cultured in hypoxia. After 48 h, cells were subjected to WST-8 assay, qRT-PCR, and immunoblotting. 

### 4.7. Luciferase Reporter Gene Analysis

The regulatory regions of the *TrkB* gene (−1880 to +42 relative to the transcription start site of exon 1a, NCBI accession no. AF410902.1) [40] and the *BDNF* gene (−629 to +281 relative to the transcription start site of exon 4, NCBI accession no. NM_170733.3; promoter IV described by Yasuda et.al) [41] were amplified using genomic DNA of SK-N-SH as a template and TrkB-F (5′-TGG GTA CCT GCC TTT ATG TAT GTG TTC ATT GGG-3′) and R (5′-TGG GTA CCG GTG ACA AAC CGT AAC TAT TCT GC-3′) and BDNF-F (5′-ATT ACG CTA GCC AGG TGA TTT TTA TGC TCC GAG G-3′) and R (5′-ATT ACG CTA GCT CGG CCC CAA AAC TCC CAC-3′) as primers, which were designed with the NCBI’s Primer-BLAST online tool (https://www.ncbi.nlm.nih.gov/tools/primer-blast). Amplified fragments were digested by KpnI for *TrkB* and NheI for *BDNF* and cloned to a corresponding multiple cloning site of a promoter-less luciferase reporter vector, pGL4.10 (Promega, Madison, WI, USA). The sequence of the clones was confirmed by a capillary sequencer, 3130xl Genetic analyzer (ABI), to be identical to the wild-type sequence found in GenBank. Sequencing primers are listed in Table 4. All procedures for construction of reporter plasmids were approved by the Toho University Administration Panel for Recombinant DNA (19-54-307).

Luciferase reporter gene analysis was done as described previously with some modification [34]. Prior to transfection with the reporter plasmids, 8 × 10^3^ SK-N-SH, TGW, or SW480 cells were plated in a well of a 96-well plate. The reporter plasmids were transected in normoxia for 24 h before adding LicA with the lipofection reagent Invitrogen Lipofectamine 3000 (Thermo Fisher Scientific, Waltham, MA, USA). The cells were cultured in hypoxia with or without LicA for 6 h. Luciferase activity was determined using the One-Glo luciferase assay system (Promega, Madison, WI, USA) and the multimode microplate reader model TriStar LB 941 (Berthold Technologies, Bad Wildbad, Germany). Two identical series of the transfected cell cultures were prepared: one for the luciferase assay and the other for counting cell density. Cell density measured by WST-8 assay was used for normalization. Promoter activity was expressed relative to values obtained under normoxic LicA-untreated conditions.

### 4.8. Statistical Analysis

Statistical significance was determined by Student’s *t*-test using the online software QuickCalcs (GraphPad, La Jolla, CA, USA). A *P* value of less than 0.05 indicated statistical significance.

## Figures and Tables

**Table 1:** Inhibitory effect of LicA on hypoxic cell growth of human cancer cell lines derived from various origins.

Cell Line	Origin	Cell Proliferation in		IC_50_, µM	
		Normoxia	Hypoxia		
SK-N-SH	Neuroblastoma	2.2 ± 0.07	1.9 ± 0.08	8.4 ± 0.3	(4)
TGW	Neuroblastoma	1.8 ± 0.07	1.4 ± 0.19	29.1 ± 1.1	(4)
GOTO	Neuroblastoma	2.0 ± 0.12	1.8 ± 0.09	31.1 ± 0.8	(4)
SW480	CRC	2.3 ± 0.06	1.8 ± 0.06	8.8 ± 0.4	(3)
SW620	CRC	3.6 ± 0.19	1.2 ± 0.06	7.0 ± 0.6	(3)
HeLa S3	Cervix carcinoma	2.2 ± 0.08	1.5 ± 0.06	10.7 ± 1.2	(3)

Normoxic and hypoxic cell proliferation of cell lines at 48 h was monitored using WST-8 assay and expressed relative fold to 0 h values. The growth inhibitory effect of LicA on cell lines under hypoxic conditions for 72 h were evaluated using WST-8 assay. IC_50_ values, determined by dose–response curves, are shown as mean ± SD derived from three or four independent experiments. The number of experiments is shown in parentheses. CRC, colorectal cancer.

**Table 2:** Inhibitory effect of LicA on hypoxic *TrkB* and *BDNF* mRNA levels of various cell lines.

Cell Line	TrkB mRNA		BDNF mRNA	
	Untreated	LicA (Inhibition, %)	Untreated	LicA (Inhibition, %)
SK-N-SH	1.00 ± 0.091	0.29 ± 0.007 (71.4) **	1.00 ± 0.067	0.71 ± 0.011 (28.7) **
TGW	3.48 ± 0.098	1.68 ± 0.119 (51.7) **	3.81 ± 0.264	1.55 ± 0.284 (59.3) **
GOTO	16.63 ± 0.429	8.74 ± 0.894 (47.4) **	N.D.	
SW480	2.49 ± 0.219	0.13 ± 0.013 (94.8) **	4.79 ± 0.406	0.16 ± 0.020 (96.6) **
SW620	0.95 ± 0.026	0.26 ± 0.017 (72.6) **	N.D.	
HeLa S3	0.09 ± 0.005	0.02 ± 0.001 (77.7) **	10.03 ± 0.250	8.66 ± 1.218 (13.7)

mRNA levels with or without 20 µM LicA were determined at 48 h in hypoxia. mRNA levels are expressed as values relative to the mRNA levels of untreated SK-N-SH for each gene. The relative values are shown as mean ± SD derived from 3 independent experiments. *P* values for differences between mRNA levels with and without 20 µM LicA in hypoxia were calculated using unpaired Student’s *t*-test (two-tail). ** *p* < 0.01. N.D., not determined.

**Table 3:** Primer sequences for qRT-PCR.

Target Gene	Sequence (5′ to 3′)		Source
	Forward	Reverse	
*TrkB*	AAGGTGGCCCAGATGCTGTC	AATGTTATGTCGCTTGATGTGCTGA	HA169993 ^1^
*BDNF*	AGTTGGGAGCCTGAAATAGTGG	AGGATGCTGGTCCAAGTGGT	This study ^2^
*NT-4*	GCGGAGGAGGTGCTGACA	GGCCAGAAAAGGGGGCAA	This study ^2^
*GAPDH*	GAAATCCCATCACCATCTTCCAGG	GAGCCCCAGCCTTCTCCATG	[35]
*18S rRNA*	CGGCTACCACATCCAAGGAA	GCTGGAATTACCGCGGCT	[36]

^1^ Primers were obtained from TakaraBio as perfect real time primers. ^2^ Primers were designed using the Primer-BLAST online tool.

**Table 4:** Sequencing primers for reporter plasmids.

Gene	Sequence (5′ to 3′)	
	Forward	Reverse
*TrkB*	F1: TCTAGACACTGTCCATGGAG	R1: ATGGGAAGGATCAAGAAACC
	F2: ATCCCTGCCTTCATGGAGTG	R2: TGTACACCAGAAGAGTCTAA
	F3: CGGAGTTTTACGTGCGTCTG	R3: TTGCGTTCTGAGTGCTCCTAGCA
	F4: AACGAGACTCCAACCCATTG	
	F5: TCAGACAAGGCTTGCAAATG	
*BDNF*	F1: GATTAACTGAGCCAGTTCTG	R1: ATTTTTTCACGTTCCCTTCG
	F2: GCGAACTAGCATGAAATCTC	R2: GCAAACACACGTATAAGCTA
Vector		R: CTTAATGTTTTTGGCATCTTCCA

Primers were designed using the Primer-BLAST online tool, except for the vector’s reverse primer; its sequence was provided by Promega.

## Abbreviations

AKT	Protein kinase B
BDNF	Brain-derived neurotrophic factor
CRC	Colorectal cancer
GAPDH	Glyceraldehyde-3-phosphate dehydrogenase
EMT	Epithelial-to-mesenchymal transition
ERK	Extracellular signal-regulated kinase
HIF-1	Hypoxia-inducible factor-1
HIF-1α	HIF-1 α-subunit
IC_50_	50% inhibitory concentration
JNK1	c-Jun N-terminal kinase 1
LicA	Licochalcone A
MAPK	Mitogen-activated protein kinase
MEK	MAPK/ERK kinase
mTOR	Mammalian target of rapamycin
NT-4	Neurotrophin-4
PI3K	Phosphatidylinositol 3-kinase
PKC	Protein kinase C
PLC- γ	Phospholipase C- γ
qRT-PCR	Quantitative reverse-transcription polymerase chain reaction
RTK	Receptor tyrosine kinase
siRNA	Small interfering RNA
TrkB	Tropomyosin-related kinase B
WST-8	2-(2-methoxy-4-nitrophenyl)-3-4-nitrophenyl)-5-(2,4-disulfophenyl)-2H-tetrazolium, monosodium
